# Effect of sweet potato endogenous amylase activation on *in vivo* energy bioavailability and acceptability of soy‐enriched orange‐fleshed sweet potato complementary porridges

**DOI:** 10.1002/fsn3.653

**Published:** 2018-04-17

**Authors:** Catherine Birungi, Agnes Nabubuya, Ivan Muzira Mukisa

**Affiliations:** ^1^ Department of Food Technology and Nutrition Makerere University Kampala Uganda

**Keywords:** amylase activation, complementary porridge, energy bioavailability

## Abstract

Energy bioavailability can be influenced by food matrix factors and processing conditions or treatments. In this study, the effects of endogenous sweet potato amylase enzyme activation and slurry solids content of soy‐enriched orange‐fleshed sweet potato (OFSP) porridges on *in vivo* energy bioavailability (energy, weight gain, and feed efficiency ratio) and porridge acceptability were determined. Fifty‐six weanling albino rats were randomly assigned to two blocks each having eight groups of seven rats. The rats were housed in individual cages in a well‐ventilated animal house. The intervention block had rats fed on activated porridges (held at 75°C for 15 min), while rats in the control block were fed on nonactivated porridges (boiled at 90–95°C for 10 min). The rats were fed for 28 days on 50 ml of porridge per rat per day. The four groups per block were each fed on porridges with varying amounts of total solids content (10%, 15%, 20%, and 25%). Weight gain, energy bioavailability, and feed efficiency ratio were determined at the end of the feeding period. Consumer acceptability of activated and nonactivated porridges at 25% solids content was determined using a nontrained human panel (*n *= 40). Activation of amylases did not significantly (*p *> .05) affect the bioavailable energy, cumulative weight gain, and feed efficiency of the rats. Increasing slurry solids content of activated and nonactivated porridges significantly (*p *< .05) increased feed efficiency ratio (−14.6 ± 11.7 to 102.3 ± 2.3), weight gain (−1.4 to 5.6 g ± 1.9 g), and bioavailable energy (702.8 ± 16.2 to 1242.8 ± 12.2 kcal). Activation of amylases reduced porridge viscosity but did not significantly influence the overall acceptability. This work demonstrates the opportunity of utilizing sweet potato amylases to facilitate the preparation of complementary porridges with appropriate viscosity and increased energy density.

## 1 INTRODUCTION

Introduction of complementary foods during infant feeding is a transitional process from breastmilk to family foods which is mainly done at a time when breast milk is no longer sufficient to meet the nutritional demands of the infant, specifically energy (Nicklaus, 2011). In sub‐Saharan Africa, most children are introduced to complementary porridges and purees (Kikafunda, Tumwine, & Walker, 2003) made from locally available raw materials, mainly starchy foods like cereals, roots, and tubers (Aderonke, Fashakin, & Ibironke, 2014). Besides the low nutrient and energy density of such complementary foods, the complementary practices in sub‐Saharan African countries are also suboptimal. In Uganda, for instance, only 14% of children aged 6–23 months receive the minimum acceptable diet of at least four different food groups (UBOS and ICF, 2017). This dire situation calls for improving infant feeding and the nutrient and energy density of complementary foods.

Complementary porridges from starchy roots, cereals, and tubers may contribute to childhood malnutrition because of their low total solids content (50–100 g/l) which are inadequate for meeting energy and nutrient requirements (Kikafunda et al., 2003; Rombo, Taylor, & Minnaar, 2001). For adequate energy and nutrient intake, porridges with at least 200 g/kg dry matter are recommended (Rombo et al., 2001). However, unless native starch is modified, swelling of starch during cooking of such products results in bulky gruels with very high viscosities making them unsatisfactory for infant feeding (Lee et al., 2008; Tawil, Viksø‐Nielsen, Rolland‐Sabaté, Colonna, & Buléon, 2012; de Carvalho, Granfeldt, Eliasson, & Dejmek, 2014). The increased viscosity of the porridges reduces energy and nutrient intake in children who have a limited gastric capacity of 30 g/kg body weight per day (Dewey, Cohen, & Rollins, 2004). In addition, once the food is ingested, net absorption of energy becomes variable and incomplete, with fecal losses accounting for 2–10% (Hall et al., 2012). Porridges that are suitable for infant feeding should preferably have viscosities in the rage of 1000–3000 cP (Rombo et al., 2001).

To increase the energy and nutrient density of complementary porridges methods such as germination, fermentation, and use of enzymes to degrade the starch have been evaluated. Several studies (Hur, Lim, Decker, & McClements, 2011; Nielsen et al., 2006) have successfully used enzyme extracts to improve bioavailability of nutrients. However, using enzyme extracts is expensive due to the high costs involved in their production (Del Pozo et al., 2012; Ncube, Howard, Abotsi, van Rensburg, & Ncube, 2012). Fortunately, endogenous amylases in foods, such as sweet potato roots, can be used in place of commercial enzymes (Hoover, 2001).

Sweet potato endogenous amylases, when subjected to appropriate conditions of temperature, pH, and time, breakdown starch into simpler sugars such as glucose and sucrose among others, which are more readily bioavailable forms of body energy (Fazekas, Szabó, Kandra, & Gyémánt, 2013; Hesam, Taheri Tehrani, & Balali, 2015). The amylase enzyme activation technique has been used to produce sweet potato purees. Sweet potato paste viscosities decrease drastically on holding at an optimum of 75°C for 10–20 min (Derde, Gomand, Courtin, & Delcour, 2012; Nabubuya et al., 2017). The decrease in sweet potato paste viscosity also allows for inclusion of more dry matter or solids in the porridges whilst maintaining low viscosity (Nabubuya et al., 2017). The endogenous amylase enzyme activation process, therefore, presents an opportunity for producing sweet potato‐based complementary porridges with increased nutrient and energy density.

Although the process of amylase activation reduces viscosity of sweet potato porridges, it is still unclear if the energy yielded is significant and more bioavailable for utilization in physiological processes of developing individuals. This study therefore investigated the effect of endogenous amylase activation on *in vivo* energy bioavailability of soy‐enriched orange‐fleshed sweet potato‐based complementary porridges using weanling male rats. It further evaluated the effect of enzyme activation on the consumer acceptance of the porridges.

## 2 MATERIALS AND METHODS

### 2.1 Raw materials

Raw materials used in the study included: Orange‐fleshed sweet potato (OFSP) roots (*Ipomea batatus*) of the NASPOT 10 variety, soy bean seeds (*Glycine max*) of Namsoy4M variety, weanling albino rats (*Rattus norvegicus*), and distilled water for formulation. All the raw materials used were obtained locally. Fresh orange‐fleshed sweet potato roots weighing 180–200 g/root were obtained from a farmer in Bombo, Luwero district, Uganda. Dried soybeans were purchased from one of the markets in Kampala District. Weanling albino rats were provided by the College of Veterinary Medicine, Animal Resources, and Biosecurity of Makerere University, Kampala, Uganda.

### 2.2 Preparation of sweet potato and soy flours

Freshly harvested orange‐fleshed sweet potato roots were washed under running water, peeled, halved longitudinally, and grated. The grated tissue was mixed thoroughly, oven dried at 40°C for 20 hr to a moisture content of 4%, and then milled into flour using a laboratory mill (Wonder mill, model 70, Korea). The sweet potato flour was sieved through a 250 μm mesh and kept in airtight containers at 4°C.

Soybean grains were sorted to remove foreign objects, washed thoroughly with clean tap water, and soaked for 6 hr so as to soften the seed coats**.** The seeds were then boiled in clean water (25 g of soy seeds: 150 ml of water) for 25 min to inactivate trypsin inhibitors. The soybean was then drained, washed with clean tap water, and separated from the hull in cold distilled water. The de‐hulled seeds were oven dried at 45°C for 20 hr and dried to a moisture content of 4% (Huang & Netravali, 2009). They were then ground into flour using a laboratory wonder mill (Model 70, Korea). Soybean flour was subsequently sieved through a 250‐μm mesh and stored in an airtight container at 4°C.

### 2.3 Methods

#### 2.3.1 Experimental layout and design

A Complete Randomised Design was used in this study. Fifty‐six male weanling albino rats were housed in individual plastic cages, with stainless wire mesh tops in a well‐ventilated animal house with 12:12 hr light/dark cycle and room temp between 22°C and 27°C. The rats were kept for seven days in the cages to get acclimatized to the conditions, during which time they fed on commercial rat pellets. The rats were then randomly assigned to two blocks representing two treatment arms: rats fed on amylase activated porridges (intervention arm) and those fed on nonactivated porridges (control arm). Each treatment arm comprised of four groups of seven rats each. The rats were fed for 28 days on 50 ml of porridge per rat per day. The four groups per block were each fed on porridges with varying amounts of total solids content (10%, 15%, 20%, and 25%) as a means of supplying varying amounts of energy. The purpose was to compare the effect of amylase activation on bioavailable energy at different energy intake levels.

#### 2.3.2 Preparation of porridges

The porridges were prepared from composite flours containing 75% OFSP and 25% soy flours. This composition was formulated in a previous study (Birungi, 2017) to meet the recommended daily allowance for carbohydrate, vitamin A, fat, minerals, and protein in weaning children. The recommended daily intakes of children aged 6–24 months are 743–1046 kcal/day, 30 g fat/day, 13 g protein/day, 95–130 g carbohydrate/day, and 300–500 μg vitamin A per day (Whitney & Rolfes, 2010). Consuming 900 ml of porridge (180 ml per feed × 5 feeds a day) of this porridge formulation at a flour rate of 20% would be sufficient to meet the recommended daily intakes for protein, carbohydrates, vitamin A, and fat (Birungi, 2017).

The porridges were prepared in two sets, at flour rates of 10%, 15%, 20%, and 25%. One set was prepared using the amylase enzyme activation technique and the other set prepared using the conventional preparation method. Amylases in the porridge were activated by heating slurries at 75°C in a water bath for 15 min (Derde et al., 2012; Nabubuya et al., 2017). The porridges were then placed on a hot plate and allowed to boil at 90–95°C for 10 min in order to deactivate the amylases and cook the porridge further. Nonactivated porridges were prepared by simply heating the slurry continuously to boiling and holding it for about 10 min at 90–95°C.

#### 2.3.3 Measuring soluble solids and viscosity of porridges

Initially, changes in soluble solids (°Brix) content of the porridges prepared using both amylase activation technique and conventional method were measured using a refractometer (model C‐3, 5901003, Barcelona) at 10 min’ intervals during porridge preparation in separate experiments. Measurements were made up to 60 min to initially confirm that amylase was activated and thus contributed to a higher release of soluble sugars at 75°C (activated) than at 95°C (nonactivation or conventional cooking of porridges).These initial experiments helped to confirm that heating at 75°C for 15 min was sufficient to achieve activation, while heating at 95°C for 10 min did not lead to activation. Final viscosity of the porridges was measured using a Brookfield viscometer (DV 11+ pro, Massachusetts, USA) under the following conditions: spindle number 63; speeds 3, 6, 12, 30, and 60 rpm.

#### 2.3.4 Proximate composition and nutrient analyses

Crude protein was analyzed by the Kjedahl method (AOAC, 2000). The nitrogen content was converted to protein using a nitrogen conversion factor of 6.25. Crude fat was analyzed by Soxhlet method (AOAC, 2000). Total carbohydrates were determined using the phenol–sulfuric acid method (Nielsen, 2010). Beta‐carotene was analyzed by the spectrophotometric method as described by Harvest Plus (Rodriguez‐Amaya & Kimura, 2004).

#### 2.3.5 Determination of bioavailable energy (BE) and feed efficiency ratio (FER)

The balance method was used to determine energy bioavailability of the porridges (Heaney, 2001). Porridges and fecal samples were first analyzed for gross energy. Fecal material to be analyzed for gross energy was collected, weighed, and stored at −20°C (Kristensen et al., 2013). Gross energy (G.E) of fecal samples and porridges was determined by adiabatic bomb calorimetry (Gerrits, Bosch, & van den Borne, 2012) using a Gallenkamp Auto bomb Calorimeter (made in UK, Serial number SG96/02/536). Rats’ weights and fecal matter were taken and recorded to the nearest 0.1 g after every four days, using a portable digital weighing scale (Acculab ECON EC‐211). Bioavailable energy was calculated as described by Jansen, Nuyens, Maertens, Van Campenhout, and Buyse (2013). Bioavailable energy was computed as the difference between energy of ingested feed and feces divided by feed intake.

Feed efficiency is a measured function of gain in body weight and feed consumed. The feed efficiency ratio (FER), in this study, was determined by obtaining the individual weights of rats gained throughout the feeding period and dividing by the average amount of feed consumed (Mundheim, Aksnes, & Hope, 2004).

#### 2.3.6 Evaluating the effect of amylase activation of soy‐enriched orange‐fleshed sweet potato porridge on consumer acceptability

Consumer acceptability of the porridges was determined by an untrained panel (*n *= 40) with members from the Department of Food Science and Nutrition, at Makerere University. Stone and Sidel (2004) recommend a panel of 25–50 members for laboratory‐based product testing. Panelists were informed that they were going to test composite porridges containing soy and OFSP, and only those who were interested and familiar with porridges were enrolled. Porridges for sensory evaluation (containing 25% solids content of amylase activated and nonamylase activated) were prepared on the day of the experiment and kept in vacuum flasks to keep hot. Porridges with 25% solids content were used as they provided the highest nutrient content and also met the viscosity requirements for feeding infants. Porridge samples were each differently coded with three‐digit numbers. About 50 ml of each porridge sample was provided to each panelist in disposable plastic cups. Panelists were asked to rinse their mouths with potable drinking water before and after assessing each sample. Porridges were rated for acceptability of taste, aroma, texture, color, thickness, after taste, overall appearance, and overall acceptability using a 9‐point hedonic scale. Sensory evaluation was carried out in individual booths under white light.

#### 2.3.7 Statistical analysis

All data are presented as means with standard deviations. The Student's t tests were used for determining differences between means of treatment groups (intervention vs. control), while ANOVA was used to compare means of response variables for different flour rates. The Tukey HSD test was sued to compare means after ANOVA. Correlations of weight gain, FER, and bioavailable energy were run. Statistical significance was accepted at *p*‐value <.05. Genstat statistical software; (4th edition developed by VSN international limited, UK) was used for statistical analysis. Statistical analysis of sensory data was done using SPSS statistical software, version 16.

## 3 RESULTS

### 3.1 Effect of heating porridges on amylase activation

**Figure 1:**
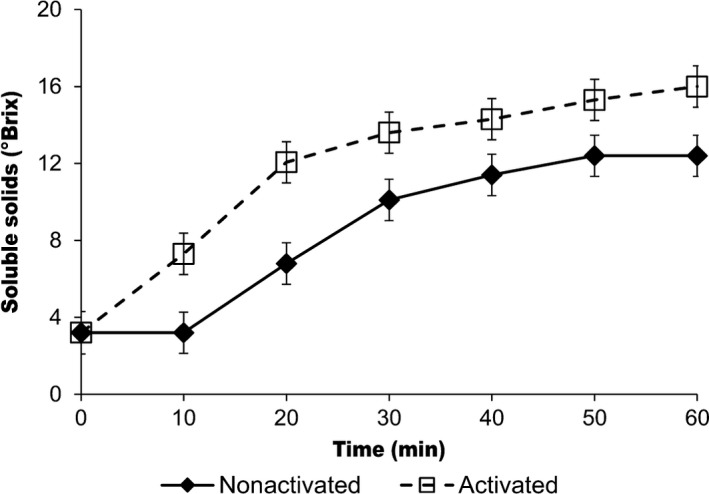
Changes in soluble solids in activated and nonactivated porridges at 20% solids content heated at 75°C for activated porridges and 90–95°C for nonactivated porridges

Soluble solids gradually increased during porridge preparation and reached a maximum after 50 min of continued heating (Figure 1). The amylase activation technique resulted in porridges with significantly higher (*p *< .05) soluble solids than the porridges prepared using the conventional method (2.4 ± 0.1 to 16.03 ± 0.06°Brix; and 2.4 ± 0.15°Brix to 12.5 ± 0.06°Brix), respectively. Heating at 75°C for 15 min was sufficient to activate amylases when compared to heating at 95°C for 10 min as the later barely yielded any changes in total soluble solids.

### 3.2 Effect of amylase enzyme activation on porridge viscosities

**Figure 2:**
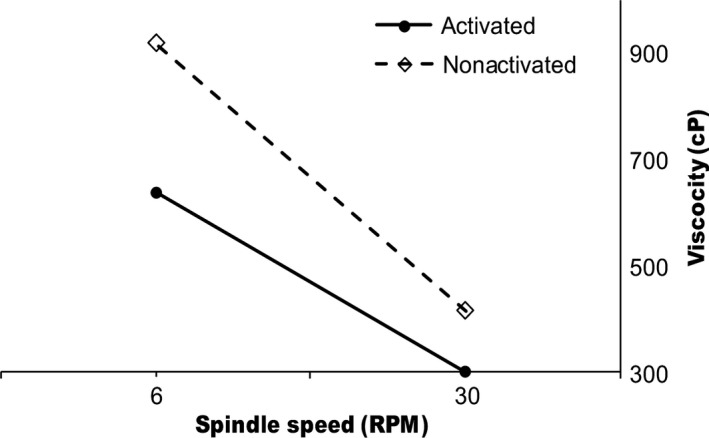
Viscosity of activated and nonactivated soy‐enriched orange‐fleshed sweet potato porridges at 20% solids content

Conventionally prepared (non activated) porridges at the same solids content had significantly (*p *< .05) higher viscosities (919.83 ± 0.1 cP, and 379.7 ± 49.0 cP) than porridges prepared using amylase activation technique (600.1 ± 69.5 cP, and 314.2 ± 29.9 cP) (Figure 2).

### 3.3 Nutrient composition and amount of experimental diets consumed

There was no significant (*p *> .05) difference observed in mean food intake of rats fed both amylase‐activated and nonactivated porridges. All porridges fed to rats contained composite flours having 25% soy and 75% orange‐fleshed sweet potato but at different levels of inclusion (10%, 15%, 20%, 25%), which ultimately resulted in differences in nutrient content (Table 1). In the experimental diets, carbohydrates (4.53 ± 0.11 g to 11.32 ± 0.27 g) contributed most to body energy, closely followed by protein (1.01 ± 1.13 g to 2.81 ± 0.29 g) and fat (0.45 ± 0.01 g to 1.12 ± 0.02 g). Mean energy values ranged from 738.35 ± 11.32 to 1332.72 ± 30.48 kcal for diets containing 10% and 25% solids content, respectively. Solids content significantly (*p *< .05) affected the nutrient composition of the porridges. The nutrient content of porridges were in the order of 10% < 15% < 20% < 25%, for all nutrients.

**Table 1:** Effect of varying solids content on average nutrient composition of experimental of porridge (100 ml)

Nutrient	Composition of nutrients at different solids content				*p*‐value
	10%	15%	20%	25%	
Crude Protein (g)	1.01 ± 1.13^a^	1.69 ± 0.18^b^	2.25 ± 0.23^c^	2.81 ± 0.29^d^	<.0001
Crude Fat (g)	0.45 ± 0.01^a^	0.67 ± 0.01^b^	0.90 ± 0.02^c^	1.12 ± 0.02^d^	<.0001
Carbohydrates (g)	4.53 ± 0.11^a^	6.79 ± 0.16^b^	9.05 ± 0.22^c^	11.32 ± 0.27^d^	<.0001
Vitamin A (μRAE)	89.3 ± 2.89^a^	133.9 ± 4.33^b^	178.6 ± 5.78^c^	223.2 ± 7.22^d^	<.0001
Gross Energy (kcal)	738.35 ± 11.32^a^	796.51 ± 1.10^b^	951.89 ± 2.39^c^	1332.72 ± 30.48^d^	<.0001

Values are means ± SD of three replicates. Means in the same row with different superscript letters (^a‐d^) are significantly different (*p *< .05).

### 3.4 Effect of amylase activation on the weight gain, efficiency ratio, and energy bioavailability of soy‐enriched orange‐fleshed sweet potato porridge *in vivo*

**Figure 3:**
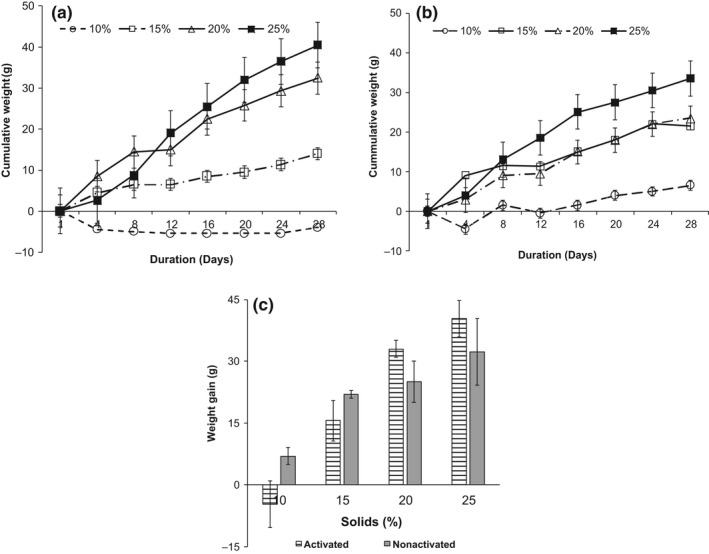
Effect of porridge solids content and amylase activation on the average cumulative weight of weanling albino rats. Key: (a) Activated; (b) nonactivated; (c) Average weight gain (g)

Consumption of both activated and nonactivated porridges resulted in cumulative weight gain in all rats (Figure 3) except for rats which consumed activated porridges with 10% solids content (Figure 3a). Feeding rats on porridges with 10% solids content led to an initial decrease in weight followed by a small or no gain in weight during the 28‐day feeding period. Cumulative weights increased in the order of 25% > 20% > 15% > 10% solids content (Figure. 3c). There were no significant differences in cumulative weight gain observed in rats fed on porridges of similar solids content prepared using either amylase activation or conventional method, but significant differences were observed in weight gain in rats fed on porridge containing different solids content (Figure. 3c).

Increase in slurry solids content resulted in significant (*p *< .05) increase in feed efficiency ratio especially in activated samples. It was also observed that preparation of porridges using amylase activation technique did not significantly (*p *> .05) affect the FER except at 25% slurry solids content (Table 2). There was a strong positive correlation between weight gain and Feed Efficiency Ratio in rats (*r *= .98).

**Table 2:** Effect of amylase activation on feed efficiency ratio (FER) of soy‐enriched OFSP porridges fed to rats

Solids content	Feed efficiency Ratio / Rat		*p*‐value across rows
	Activated	Nonactivated	
10%	−14.56 ± 11.77^ax^	16.93 ± 2.88^ay^	.028
15%	31.44 ± 15.11^bx^	60.18 ± 19.11^bx^	.129
20%	66.19 ± 5.61^cx^	58.50 ± 8.65^bx^	.114
25%	102.29 ± 2.25^dx^	69.52 ± 22.39^by^	.047
*p*‐values along columns	<.0001	.003	

Values are means ± SD (*n *= 56). Means in the same column followed by different superscripts (^a‐d^) and means in the same rows followed by different superscript (^x‐y^) are significantly different (*p *< .05).

Bioavailable energy ranged from 702.8 to 1242.4 kcal and 705 to 1252.9 kcal/rat in activated and nonactivated porridges, respectively. Activation did not significantly increase (*p *> .05) bioavailable energy, whereas increasing solids content resulted in significantly (*p *< .05) higher levels of bioavailable energy (Table 3).

**Table 3:** Effect of amylase activation on bioavailable energy of soy‐enriched OFSP porridges

Solids content	Average bioavailable energy (kcal /rat)		
	Non amylase activated	Amylase activated	*p*‐value across rows
10%	702.812 ± 16.2^ax^	705.005 ± 24.4^ax^	.792
15%	738.718 ± 3.06^bx^	720.090 ± 21.23^bx^	.096
20%	883.431 ± 3.95^cx^	921.57 ± 34.7^cx^	.057
25%	1242.805 ± 12.15^dx^	1252.9 ± 0.596^dx^	.126
*p*‐value along columns	<.0001	<.0001	

Values are means ± SD. (*n *= 56). Means along the columns followed by different superscripts (^a‐d^) and means in the same row followed by different superscripts (^x‐y^) differ significantly (*p *< .05).

### 3.5 Effect of amylase enzyme activation on porridge acceptability

Both activated and nonactivated porridges (containing a total solids content of 25%) were accepted by the panelists (Table 4). Activation of the porridges had no significant (*p *> .05) effect on the acceptability scores of most porridge attributes except thickness. The thickness of the nonactivated porridges was significantly (*p *< .05) more acceptable than that of the activated porridges.

**Table 4:** Effect of amylase activation on the acceptability of soy‐enriched OFSP‐weaning porridge

Attributes	Amylase activated Porridge	Nonamylase activated Porridge	*p*‐value
Color	6.00 ± 1.41^a^	6.05 ± 1.76^a^	.922
Aroma	6.35 ± 1.53^a^	6.50 ± 1.67^a^	.769
Texture	7.05 ± 1.82^a^	7.20 ± 2.07^a^	.809
Thickness	6.15 ± 1.93^b^	7.35 ± 1.57^a^	.037
Taste	5.70 ± 1.92^a^	5.67 ± 1.84^a^	.934
After taste	5.80 ± 1.77^a^	5.80 ± 1.67^a^	1.000
Overall appearance	6.50 ± 1.43^a^	6.45 ± 1.28^a^	.908
Overall acceptance	6.50 ± 1.85^a^	6.25 ± 1.83^a^	.670

Values are means ± SD (*n *= 40). Means in the same row followed by different superscript letters (^a, b^) differ significantly (*p *< .05). A nine‐point hedonic scale anchored as follows was used to score the acceptability: 1 =  “dislike extremely,” 2 =  “dislike very much,” 3 =  “dislike moderately,” 4 =  “dislike slightly,” 5 =  “neither like nor dislike,” 6 =  “like slightly,” 7 =  “like moderately,” 8 =  “like very much,” and 9 =  “like extremely.”

## 4 DISCUSSION

The bioavailable energy in rats, resulting from consuming amylase activated and nonactivated porridges at the same solids content, was not significantly different. These findings reveal that although amylase activation increases the amount of simple sugars in food (Derde et al., 2012; Kaur & Vyas, 2012), the rate at which these sugars were absorbed from the GIT was most likely not significantly different, when compared to porridges of similar solids content prepared with no amylase activation. This may suggest that when amylase activated and nonamylase‐activated porridges are subjected to heating conditions, the starch granules are gelatinized, hence markedly increasing susceptibility to both inherent sweet potato and gastrointestinal tract amylolytic degradation, respectively (Svihus, Uhlen, & Harstad, 2005). In fact, Holm, Lundquist, Björck, Eliasson, and Asp (1988) found a correlation of 0.96 between extent of gelatinization and digestion rate, indicating that the relationship is close to linear, for pure starch. This implies that feeding on either porridges at the same solids content may result in similar energy intakes. However, as activation produces less viscous porridges, then higher amounts of solids can be packed into the porridge. By adding more dry matter to the porridge, not only does the bioavailable energy increase but also the overall nutrient density. This, therefore, enables the provision of more nutrients and energy to the infant.

Feed efficiency is a measured function of gain in body weight and feed consumed. It may also be termed as the rate at which food is effectively utilized to contribute to the weight gain of subjects who consume it (Mundheim et al., 2004). Amylase activation in this study significantly increased the feed efficiency. One study (Tsou & Hong, 1989) suggested that a low feed efficiency of sweet potato diet is due to a lower digestibility of nutrients. The study further mentioned that cooking is an effective method for improving digestibility and overall feed efficiency (Tsou & Hong, 1989). It is also well documented that raw potato starch is poorly digested by rats (Holm et al., 1988). In this study, both porridge treatments were subjected to heat and thoroughly boiled before they were fed to the rats thus ensuring that porridges were digestible.

Rats fed porridges containing 10% initially lost and hardly gained any weight most likely because the solids content could not provide sufficient nutrients and energy that are required for optimal growth and thus weight gain. Rats fed on amylase‐activated porridges gained relatively more cumulative weight than rats on nonactivated porridges. The increase in weight and feed efficiency ratio in rats fed on amylase activated porridges in the study may have resulted from a reduced thermic effect of food following ingestion (Tester, Qi, & Karkalas, 2006). Thermic effect of food (TEF) is the amount of energy expended during digestion. TEF contributes to total energy expenditure (EE), because of increases in metabolic rate as the body tries to break down food into absorbable and utilizable components (Binns, Gray, & Di Brezzo, 2015). *Ex vivo* amylase activation leads to increased amounts of simple sugars following starch hydrolysis (Tester et al., 2006), hence facilitating absorption and thus reducing the thermic effect requirement of food. Therefore, although both activated and nonactivated porridges yield similar amounts of bioavailable energy, the sparing effect of *ex vivo* amylase activation on thermic effect could have contributed to observed differences in weight gain.

The study revealed a close relationship between cumulative weight gain and energy bioavailability: high cumulative weights correlated with high bioavailable energy. Studies reveal that food nutrients are transformed *in vivo* following absorption to substrates that can produce metabolically useful energy (Lee et al., 2015; Sitrin, 2014). This transformation is done by intestinal enzymes, and the energy produced may either be stored or used to conduct biological processes such as growth and body maintenance (French, Epstein, Jeffery, Blundell, & Wardle, 2012). Depending on body weight as an indicator, bioavailable energy may be in positive or negative balance, but it varies within a 24‐hr period and across the life span because of individual body energy demands (Speakman & Westerterp, 2010).

In this study, panelists preferred the thickness of nonactivated porridge to activated porridges. The presence of activated endogenous amylases in the porridge was responsible for the low viscosity of the porridge. Thaoge et al. (2003) also stated that amylase enzymes hydrolyze starch to dextrin and maltose which reduces the viscosity of porridges. Nonamylase activated porridges, on the contrary, had more starch, which swelled up during heating yielding more viscous porridges. The acceptability of enzyme‐activated porridges could be enhanced by increasing the dry matter content, which would in effect further boost their nutrient density.

## 5 CONCLUSION

Activation of endogenous amylases in porridge formulations may have minimal or no effects on product acceptability and *in vivo* energy bioavailability. In this study, activation only significantly reduced the acceptability for thickness indicating that consumer prefers thick to thin porridge. Increasing the solids content of porridges has a greater effect on bioavailable energy and weight gain than activation alone. For maximum gain in body weight, nutrient intake and bioavailable energy, soy‐enriched orange‐fleshed sweet potato porridge with solids content of ≥25% and above need to be prepared. Given that amylase activation and nonactivation at similar solids content do not produce significant differences in bioavailable energies, amylase‐activated porridges seem to be a good option for preparing complementary porridges containing solids content ≥25%. Amylase activation allows for incorporation of more solids content (thus obtaining higher nutrient densities) whilst yielding products with recommended viscosity for infant feeding. Policies on therapeutic management of under nutrition‐related problems in resource‐limited settings where OFSP is being promoted should consider promoting the use of enzyme‐activated porridges. On the contrary, nonamylase‐activated porridges containing ≥25% solids are too thick for children <2 years. However, even with the knowledge gained from this study, it is still important to note that animal studies are only able to lay the groundwork for research in humans. In order to draw conclusions for human populations, human studies will be required.

## CONFLICT OF INTEREST

Authors declare that there is no conflict of interest.
